# The use of nanovibration to discover specific and potent bioactive metabolites that stimulate osteogenic differentiation in mesenchymal stem cells

**DOI:** 10.1126/sciadv.abb7921

**Published:** 2021-02-26

**Authors:** Tom Hodgkinson, P. Monica Tsimbouri, Virginia Llopis-Hernandez, Paul Campsie, David Scurr, Peter G. Childs, David Phillips, Sam Donnelly, Julia A. Wells, Fergal J. O’Brien, Manuel Salmeron-Sanchez, Karl Burgess, Morgan Alexander, Massimo Vassalli, Richard O. C. Oreffo, Stuart Reid, David J. France, Matthew J. Dalby

**Affiliations:** 1 Centre for the Cellular Microenvironment, Institute of Molecular, Cell and Systems Biology, College of Medical, Veterinary and Life Sciences, University of Glasgow, Glasgow G12 8QQ, UK.; 2 Tissue Engineering Research Group, Department of Anatomy and Regenerative Medicine, Royal College of Surgeons in Ireland, Dublin D2, Ireland.; 3 SUPA Department of Biomedical Engineering, University of Strathclyde, Glasgow G1 1QE, UK.; 4 School of Pharmacy, The University of Nottingham, Nottingham NG7 2RD, UK.; 5 Centre for the Cellular Microenvironment, Division of Biomedical Engineering, School of Engineering, University of Glasgow, Glasgow G12 8LT, UK.; 6 School of Chemistry, College of Science and Engineering, University of Glasgow, Glasgow G12 8QQ, UK.; 7 Bone and Joint Research Group, Centre for Human Development, Stem Cells and Regeneration, Institute of Developmental Sciences, University of Southampton, Southampton SO16 6YD, UK.; 8 Glasgow Polyomics, College of Medical, Veterinary and Life Sciences, University of Glasgow, Switchback Rd., Bearsden, Glasgow G61 1BD, UK.

## Abstract

Bioactive metabolites have wide-ranging biological activities and are a potential source of future research and therapeutic tools. Here, we use nanovibrational stimulation to induce osteogenic differentiation of mesenchymal stem cells, in the absence of off-target, nonosteogenic differentiation. We show that this differentiation method, which does not rely on the addition of exogenous growth factors to culture media, provides an artifact-free approach to identifying bioactive metabolites that specifically and potently induce osteogenesis. We first identify a highly specific metabolite, cholesterol sulfate, an endogenous steroid. Next, a screen of other small molecules with a similar steroid scaffold identified fludrocortisone acetate with both specific and highly potent osteogenic-inducing activity. Further, we implicate cytoskeletal contractility as a measure of osteogenic potency and cell stiffness as a measure of specificity. These findings demonstrate that physical principles can be used to identify bioactive metabolites and then enable optimization of metabolite potency can be optimized by examining structure-function relationships.

## Teaser

Osteogenic activity metabolites can be identified in nanovibrational culture and have their specificity and potency enhanced.

## INTRODUCTION

Metabolites, the substrates and products of metabolism, are known to have wide-ranging functions in cells and organisms. In stem cell research, there is considerable interest in using metabolites as biomarkers of growth and differentiation, for example, to provide measurement of batch process in the manufacturing of cell therapies (*1*). However, there is emerging evidence that bioactive metabolites (which have biological activity and are also known as activity metabolites) can be identified that drive and regulate cellular processes, such as differentiation (*2*–*5*). This is logical as metabolites feed into and contribute to a wide range of biochemical processes that can influence cell behaviors (*6*).

Bioactive metabolites have the potential to become important research tools that can be used to control the differentiation and activity of cells. For example, they could be used to stimulate stem cell differentiation, removing the need to use complex media formulations, which often contain powerful growth factors or corticosteroids that can produce off-target and desired differentiations (*2*). We note that bioactive metabolites might also lend themselves to chemical modification to enhance their specificity and potency.

A critical tool in the discovery of candidate bioactive metabolites is development of methods that can control stem cell behaviors without changing media formulations. This is important as changing media to, for example, control differentiation program in one well, while maintaining phenotype in the control well will add artifact to metabolome surveillance (*2*). Thus, physical approaches, such as use of biomaterials or mechanical forces, will be important in identification of bioactive metabolites.

We have previously developed a nanomechanical bioreactor that drives the differentiation of mesenchymal stem cells (MSCs) toward the osteogenic (bone) lineage in a highly specific manner (*7*). This bioreactor uses the reverse piezo effect to turn electrical input into mechanical movement, with piezo active ceramics placed under a ferrous actuating top plate. This top plate vibrates with a 60-nm peak-to-peak amplitude, which produces a 30-nm displacement of the plate at 1000 Hz. The bioreactor was developed in response to observations of cells vibrating their adhesions on the nanoscale (*8*) as a way to see whether vibrating the adhesive surface back at the cells could change phenotype. While fast, 1000-Hz, membrane vibrations, termed flickering, are observed in erythrocytes, nucleated cell vibrations tend to be slower, in the 0.5 to 10 s of hertz range (*9*–*12*). Our optimal vibration of 1000 Hz is clearly substantially faster than this. However, it is known that hydrated collagen, as in the bone, requires kilohertz-range stimulus for piezoelectric effect to be observed (*13*) and that piezoelectricity in the bone can stimulate osteoblast activity (*14*).

We have used the bioreactor to differentiate MSCs in two-dimensional (2D) (*15*) and 3D (*7*) culture toward the osteogenic lineage without the use of specialized media; the cells are cultured in the same media as unstimulated controls. Typically, MSCs are stimulated to undergo osteogenic differentiation through the use of osteogenic media (OGM), which contains a cocktail of dexamethasone (a corticosteroid), ascorbic acid, and glycerophosphate (*16*). However, dexamethasone also drives MSCs to undergo adipogenic differentiation (*16*) and thus acts in a nonspecific manner.

In this study, we demonstrate that the nanovibrational stimulation of MSCs can be used to identify highly specific bioactive metabolites that control MSC osteogenic differentiation. To achieve this, we used Stro1^+^ MSCs isolated from the human bone marrow (*17*). We note that the term “mesenchymal stem (stromal) cell” is now widely used and has come to often represent an adherent fibroblastic population of cells, even those that are not stem cells based on rigorous criteria (*18*). For avoidance of confusion, in this study, we use the term MSCs to refer to skeletal stem cells—a clonogenic population of nonhematopoietic bone marrow stromal cells that can recreate the cartilage, bone, hematopoiesis-supporting stroma, and marrow adipocytes on the basis of in vivo transplantation studies (*18*). We then used nanovibrational MSC culture and mass spectrometry to identify the endogenous steroid, cholesterol sulfate, as a specific osteoinducer. By then examining structure-function relationship between cholesterol sulfate and dexamethasone, we also showed that we could identify fludrocortisone acetate as being both a specific and highly potent osteoinducer. Our findings thus demonstrate how physical principles can be used to control cell responses and behaviors in discovery pipelines.

## RESULTS

### 2D and 3D osteogenesis

**Fig. 1:**
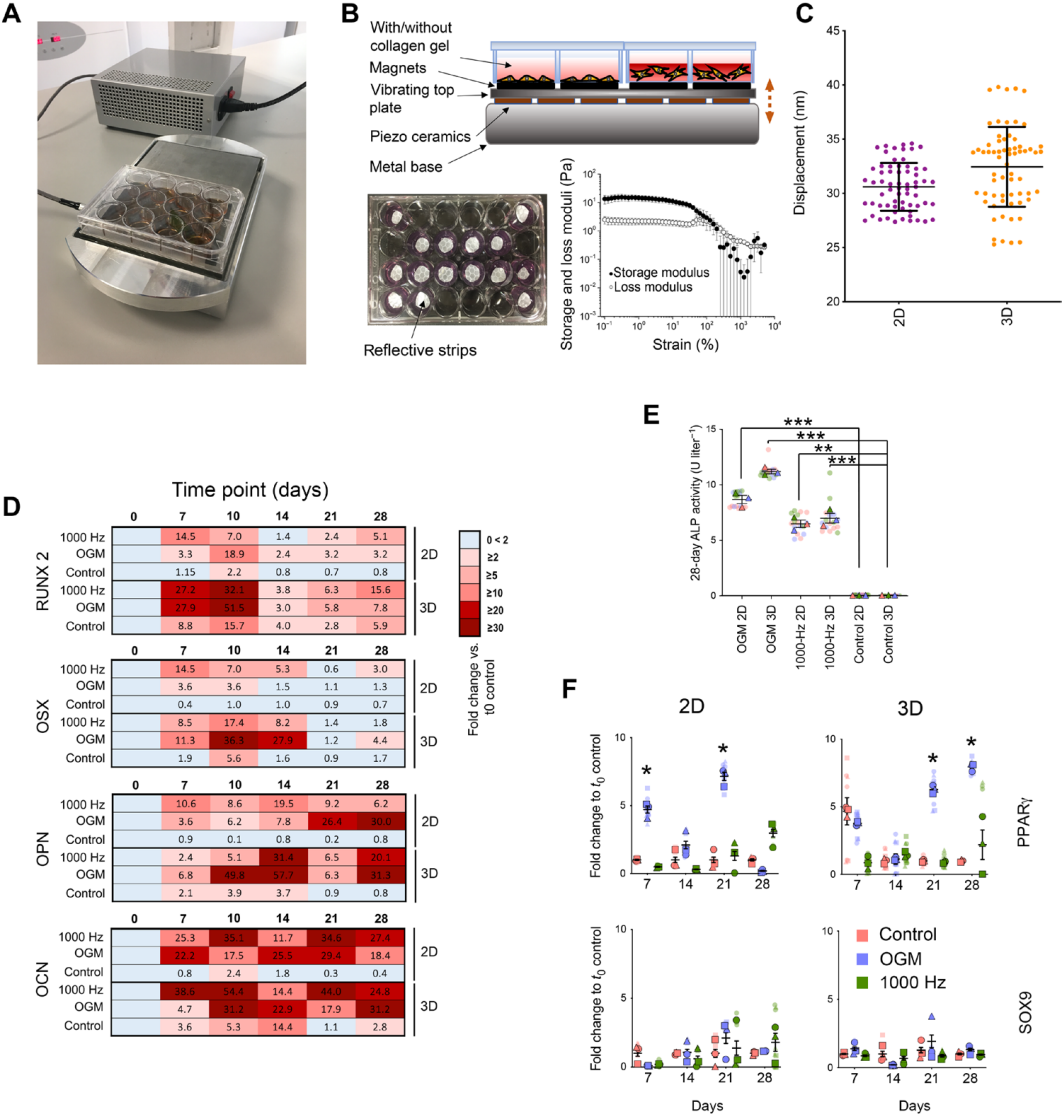
Nanovibration drives osteogenic differentiation in 2D and 3D MSC cultures. (**A**) The nanovibrational bioreactor; cultureware is magnetically attached to a piezo-driven vibrational plate. (**B**) Cross-sectional drawing depicting 2D and 3D culture setups (3D culture; type I collagen gel; *G*′ ~ 13.7 Pa and *E* ~ 40 Pa). (**C**) Laser interferometry; mean vibration amplitude is within 2.5 nm of the 30-nm target (13 gels measured, *n* = 65 measurements). (**D**) Heatmap of osteogenic marker up-regulation in MSCs cultured over 28 days with nanovibration (1000 Hz) or OGM [quantitative reverse transcription polymerase chain reaction (qRT-PCR)], relative to day 0 and time-matched unstimulated controls (*d* = 1, *r* = 4, and *t* = 3, where *d* is the number of donors, *r* is the number of replicate wells, and *t* is the technical replicates). (**E**) Alkaline phosphatase (ALP) activity assay showed significant increases in 1000-Hz and OGM groups, compared to unstimulated day 28 controls, *d* = 3, *r* = 2, and *t* = 3 (different colors indicate donors). (**F**) qRT-PCR of chondrogenic (*SOX9*) and adipogenic [peroxisome proliferator–activated receptor γ (*PPAR*γ)] expression showed increased PPARγ expression in OGM groups compared to unstimulated day 0 controls, *d* = 3, *r* = 3, and *t* = 3 (different shapes indicate donors). Significance values calculated using Kruskal-Wallis with Dunn’s multiple comparison test. All results are means ± SEM. *P < 0.05, **P < 0.01, and ***P < 0.001. Larger versions of (E) and (F) are available in fig. S3. Photograph credits: Paul Campsie, University of Strathclyde.

Cell culture plates were firmly attached to the bioreactor (Fig. 1A) by magnets (Fig. 1B), enabling displacement to travel into the cell plates while allowing for their easy maintenance. To facilitate calibration, we placed reflective strips into the bottom of the plates’ wells, which were used for 2D culture. For 3D cultures, we used type I collagen hydrogels (stiffness, 40 Pa; as measured by rheology) and placed the strip on top of the hydrogel (Fig. 1B and fig. S1). Thirty-nanometer displacements were measured using laser interferometry for both 2D and 3D conditions (Fig. 1C). We used collagen for our experiments as it has low stiffness, below the 30- to 40-kPa stiffness required to drive MSC osteogenesis (*19*, *20*), and is biocompatible. It also sticks to the sides of the cell culture plates, providing mechanical integration with the plate. As a hydrogel, it is also incompressible (*21*), which means that it acts as a solid volume when vibrated in a contained environment, such as the wells of a culture plate; this provides good fidelity of vibration throughout its volume (*7*).

Before using this experimental setup to identify bioactive metabolites, we first checked that the nanovibrational stimulation (1000 Hz) of MSCs under 2D and 3D culture conditions specifically stimulated osteogenesis. To do so, Stro-1–selected skeletal MSCs were seeded in 2D culture or within collagen gels for 3D culture and were nanovibrated for up to 28 days. The expression of an early osteogenic marker, runt-related transcription factor 2, (*RUNX2*), a mid-stage marker, osterix (*OSX*), and two late-stage osteogenic markers, osteopontin (*OPN*) and osteocalcin (*OCN*), was assessed by quantitative reverse transcription polymerase chain reaction (qRT-PCR) and the expression profiles of stimulated cells (1000 Hz and OGM) normalized to those of unstimulated controls at day 0. The effect of culture on the expression of osteogenic markers in unstimulated MSCs was also assessed over 28 days and compared to 1000 Hz– and OGM-stimulated MSCs. MSCs cultured in either 2D or 3D and stimulated by 1000 Hz or by OGM produced similar patterns of osteogenic marker expression, with 3D culture conditions typically producing higher levels of osteospecific differentiation relative to 2D conditions (Fig. 1D and fig. S2). Alkaline phosphatase (ALP) protein levels also showed a similar trend with both 1000-Hz and OGM stimulation enhancing osteoblastic differentiation (Fig. 1E). Using this same approach, we assessed the expression of adipogenesis [peroxisome proliferator–activated receptor γ (*PPAR*γ)] and chondrogenesis [SRY-box transcription factor 9 (*SOX9*)] markers by qRT-PCR. In OGM-stimulated cultures, *SOX9* was not expressed; however, we observed, as expected (*16*), increased *PPAR*γ expression under both 2D and 3D culture conditions (Fig. 1F). In 1000 Hz–stimulated cultures, no evidence of off-target differentiation was observed (Fig. 1F), demonstrating that nanovibrational stimulation specifically stimulates osteogenic differentiation in the absence of defined media.

### Surveying metabolic changes in OGM-treated and nanovibrated MSCs

**Fig. 2:**
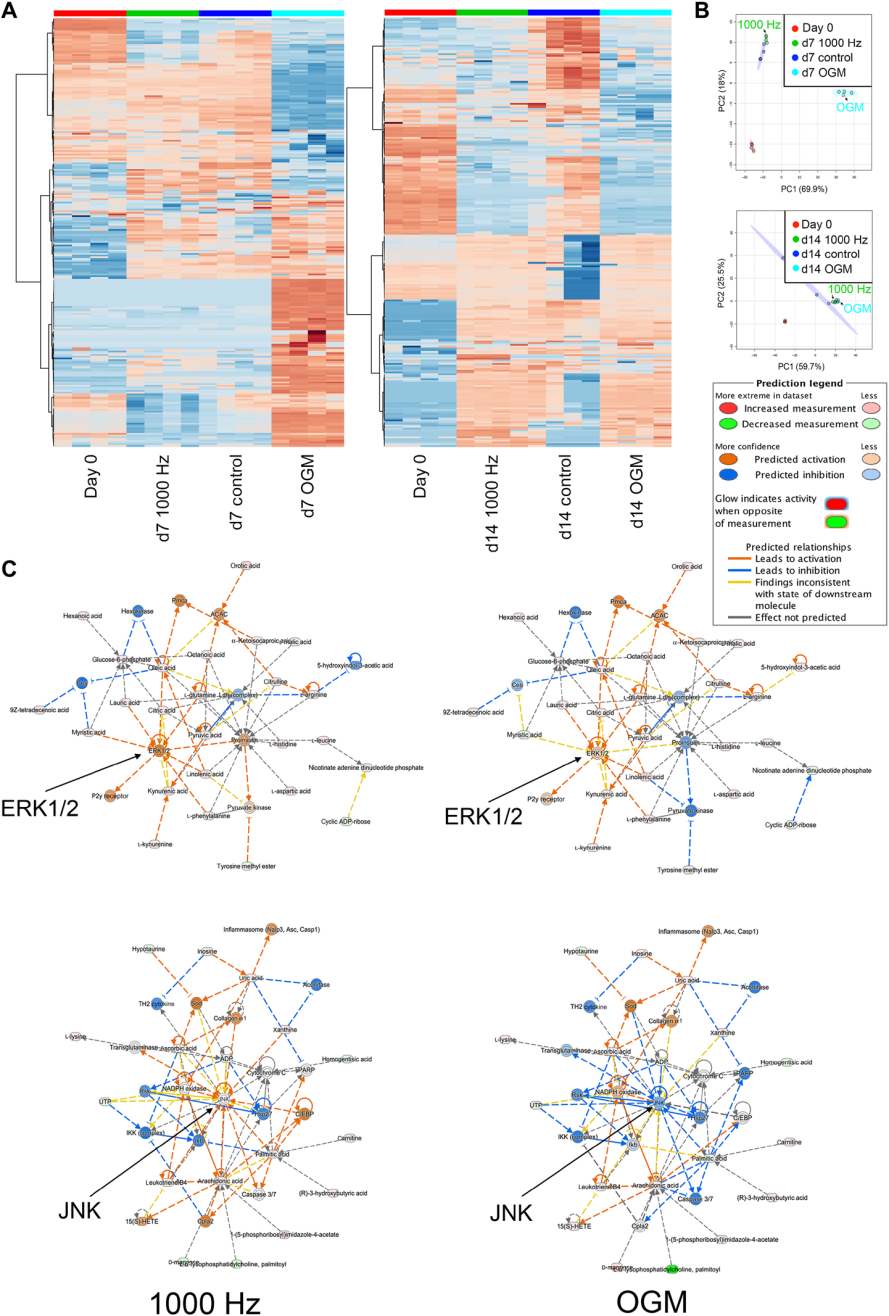
Metabolic profiles of MSCs in 3D culture for 7 days with OGM or nanovibration. (**A**) Heatmap (red, up-regulations; blue, down-regulations) and (**B**) principal components (PC) analysis depicting differences in nanovibration and OGM metabolic profiles during MSC differentiation. Metabolic profiles of MSCs induced by nanovibrational stimulation show a greater similarity to unstimulated controls at day 7 (d7) of culture and to OGM-stimulated cells at day 14 (d14). (**C**) Metabolic profiles were compared by Ingenuity Pathway Analysis (IPA). The analysis uses a curated literature database to plot metabolite interactions linked to abundance (red, increased measurement; green, decreased measurement). Further, it links groups of metabolites to biochemical pathways, such as extracellular signal–related kinase 1/2 (ERK1/2) and c-Jun N-terminal kinase (JNK), predicting pathway up-regulation and down-regulation (orange, predicted activation; blue, predicted inhibition). The metabolic profiles of nanovibrated cells lead to stronger predicted activation of ERK1/2 relative to the metabolic profiles of cells induced by OGM. In contrast, the metabolic profiles of OGM-stimulated cells lead to stronger predicted down-regulation of JNK, while those of nanovibrated cells predict JNK’s activation rather than inhibition. Networks are built from *d* = 1 and *r* = 4. ACAC, acetyl-CoA carboxylase alpha; ADP, adenosine 5’-diphosphate; Asc, Apoptosis-associated speck-like protein containing a CARD; Casp1, caspase 1; C/EBP, CCAAT/enhancer binding protein; Ces, carboxylesterase; Cpla2, Cytosolic phospholipases A 2; 15(S)-HETE,15-Hydroxyeicosatetraenoic acid; Hsp27, Heat shock protein 27; Ldh, lactate dehydrogenase; Ikb, nuclear factor of kappa light polypeptide gene enhancer in B-cells inhibitor; IKK, inhibitor of nuclear factor κB kinase.; NADPH, reduced form of nicotinamideadenine dinucleotide phosphate; Nalp3, NLR family pyrin domain containing 3; Pmca, plasma membrane Ca^2+^ ATPase; P2y, purinoceptor; Rsk, ribosomal s6 kinase; Sod, superoxide dismutase; TH2, Type 2 helper T cells; UTP, uridine 5’-triphosphate. (Please note that larger versions of principal components analysis plots and pathway maps are available in fig. S4).

We next used liquid chromatography–mass spectrometry (LC-MS) (*22*) to survey metabolic changes in MSCs cultured in 3D, as this culture condition is more physiological and produced the greatest changes in marker expression. We observed metabolite changes at days 7 and 14 of culture with or without nanostimulation and with OGM. We focused on the lipid compartment (Fig. 2, A and B) as the metabolite grouping with the most abundant change. Our data and principal components analyses showed that at day 7, the lipidomes of 1000 Hz–stimulated MSCs and of unstimulated day 0 and unstimulated day 7 controls were similar (Fig. 2A) but distinct along principal component 2 (Fig. 2B), while the lipidomes of OGM-treated MSCs showed large-scale changes. By day 14 of culture, both nanovibrated and OGM-stimulated MSCs had lipid trends that grouped together and that were divergent from unstimulated day 0 and unstimulated day 14 control cells (Fig. 2, A and B).

On the basis of our qRT-PCR data (see Fig. 1D), we selected day 7 as the time point at which osteogenesis has been initiated (*23*, *24*) and at which clear changes were evident in the metabolic profiles of nanovibration-stimulated versus OGM-stimulated cells. We used Ingenuity Pathway Analysis (IPA) to analyze the day 7 data to build predictive pathways linked to mitogen-activated protein kinase pathways, which are regularly linked to MSC osteogenesis, namely, extracellular signal–regulated kinase 1/2 (ERK1/2) and c-Jun N-terminal kinase (JNK) (*25*–*28*). This analysis showed that for both 1000 Hz– and OGM-stimulated cells, the metabolite expression pattern predicted an up-regulation of ERK1/2 (Fig. 2C). By contrast, the predictions for JNK were divergent. The metabolite expression pattern of 1000 Hz–stimulated cells predicted the up-regulation of JNK, while that of OGM-stimulated cells predicted JNK’s down-regulation (Fig. 2C). Together, these data indicate that metabolism differs between 1000 Hz– and OGM-stimulated cells that are undergoing osteogenic differentiation and that a more targeted differentiation can be achieved with nanostimulation.

### Identification of activity metabolites

**Fig. 3:**
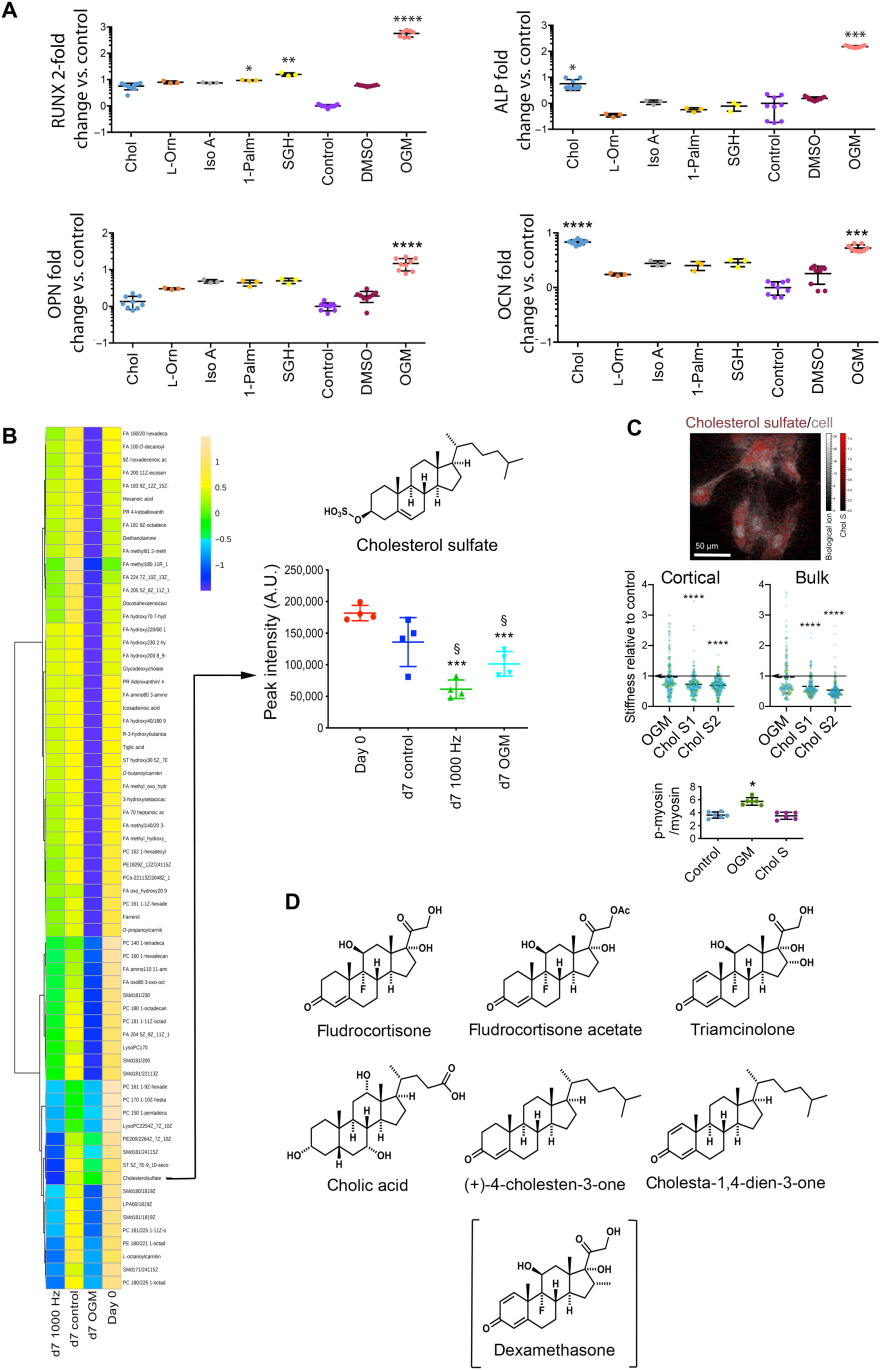
Screening metabolites for osteogenic bioactivity in MSCs. (**A**) Differential osteogenic gene expression [*RUNX2* (day 7), *ALP* (day 14), and *OPN/OCN* (day 21)] in MSCs supplemented with metabolites depleted during OGM and nanovibration osteogenic differentiation, 1 μM cholesterol sulfate up-regulated late-stage markers, *ALP* and *OCN*. Means ± SEM, versus unstimulated time-matched controls, *d* = 1, *r* = 3, and *t* = 3. Chol, cholesterol sulfate; l-Orn, l-ornithine monohydrochloride; Iso A, isonicotinic acetate; 1-Palm, 1-palmitoyl-*sn*-glycero-3-phosphocholine; SGH, sodium glycocholate hydrate; DMSO, dimethyl sulfoxide control. (**B**) Heatmap comparing MSC lipid metabolism (columns indicate means; *d* = 1 and *r* = 4). OGM and nanovibration osteogenic differentiation depleted cholesterol sulfate. Means ± SEM, *d* = 1, *r* = 3, and *t* = 3. ac, acid; FA, fatty acid; PR, prenol lipid. (**C**) OrbiSIMS image of cholesterol sulfate within MSCs, *d* = 1, *r* = 3, and *t* = 3. MSC stiffness (Young’s modulus, nanoindentation). Chol S1 and Chol S2 indicate 1 and 2 μM cholesterol sulfate, respectively. Cholesterol sulfate reduces cortical and bulk cell stiffness, *d* = 2, *r* = 2, and *t* ≥ 100; colors denote donors, and lines denote means (arrows indicate control mean). In-cell Western of p-myosin/total myosin. Cholesterol sulfate reduced intracellular tension, means ±SD, *d* = 1, *r* = 4, and *t* = 1. (**D**) Molecular structures of selected molecules combining elements of cholesterol sulfate and dexamethasone. (A and C) Kruskal-Wallis with Dunn’s multiple comparison test. (B) One-way analysis of variance (ANOVA) with Tukey multiple comparison test. *P < 0.05, **P < 0.01, ***P < 0.001, and ****P < 0.0001. Larger version of heatmap is shown in fig. S5. A.U., arbitrary units.

On the basis of our results, we hypothesized that we could identify bioactive metabolites with osteogenesis-inducing activity by looking at day 7 mass spectrometry data because, at this time point, the metabolite changes in 1000 Hz–stimulated cells are likely to be linked to osteogenic differentiation. We also hypothesized that corroborative changes present in the less-specific OGM data would point us to the essential metabolic changes that accompany osteogenic commitment. To select metabolites, we looked for those that were depleted in 1000 Hz– and OGM-stimulated MSC cultures, relative to unstimulated day 0 and unstimulated day 7 control MSCs [raw data available at (*29*)]. In this way, we identified five candidate metabolites, which were each tested for their ability to induce osteogenic marker gene expression by qRT-PCR at day 7 (*RUNX2*), day 14 (*ALP*), and day 21 (*OPN* and *OCN*) culture with MSCs (at 1 μM concentration; fig. S6) without nanovibrational stimulation. From these candidate bioactive metabolites, three were able to induce the increased expression of osteogenic markers when unstimulated, with cholesterol sulfate producing the most robust osteogenic response (Fig. 3A). Cholesterol sulfate is a cell membrane–associated sterol lipid that is considered to provide structural support but that is also a regulatory molecule associated with the transforming growth factor–β (TGFβ) family (*30*, *31*). Members of the TGFβ superfamily, such as bone morphogenetic protein 2 (BMP2), have known osteogenic properties (*32*–*34*).

Figure 3B shows the metabolite lipid compartment of MSCs after 7 days of 3D culture, and the depletion of cholesterol sulfate in 1000 Hz– and OGM-stimulated MSCs compared to unstimulated day 7 and unstimulated day 0 controls. To probe the spatial distribution of cholesterol sulfate in cells, we turned to the recently reported mass spectrometry technique, 3D OrbiSIMS (*35*). This technique uses a hybrid mass spectrometer approach, comprising time-of-flight–secondary ion mass spectrometry (TOF-SIMS) and orbitrap mass spectrometry. While TOF-SIMS rapidly gives spatial information, orbitrap provides accurate small-molecule detection at specific locations. Using this technique, we observed cholesterol sulfate to be localized within cells fed with cholesterol sulfate–supplemented media, after 72 hours of culture (Fig. 3C and fig. S7). As cholesterol sulfate is membrane associated and was retained in cells, we hypothesized that it would alter the stiffness of the MSCs. Using nanoindentation to assay cell mechanics, we observed a significant decrease in Young’s modulus (*E*) in both the cell cortical region (first 270 nm) and the bulk cell (first 670 nm) (Fig. 3C). This indicated that cholesterol sulfate decreases cell stiffness.

We next assessed phosphorylated myosin (p-myosin), specifically pSer^19^, as Rho-associated protein kinase, which is involved in cytoskeletal contraction (*36*), phosphorylates myosin at this position. In cholesterol sulfate–treated cells, we observed that while OGM treatment increased MSC cytoskeletal tension, cholesterol sulfate treatment did not cause any change from control (Fig. 3C). By observing fluorescently stained actin in MSCs treated with cholesterol sulfate, we found these cells to have similar actin stress fiber organization to control, while, again, OGM-treated cells displayed more prominent stress fibers indicative of increased cytoskeletal tension (fig. S8).

Although the specificity of cholesterol sulfate for osteogenic differentiation was high, we were curious to see whether the potency of this effect could be improved. The widespread use of the synthetic glucocorticoid dexamethasone to induce differentiation (*37*), combined with our observation that cholesterol sulfate (*30*, *31*) also induces osteogenesis in MSCs, prompted us to focus on the steroid scaffold. We therefore looked for molecular structures of small molecules that combined structural elements of cholesterol sulfate and dexamethasone and screened those that did in MSCs. To do so, we elected to screen a library of natural and synthetic steroids (Fig. 3D) to see whether we could improve on the potency and specificity of cholesterol sulfate response.

### Fludrocortisone acetate shows enhanced potency and specificity

**Fig. 4:**
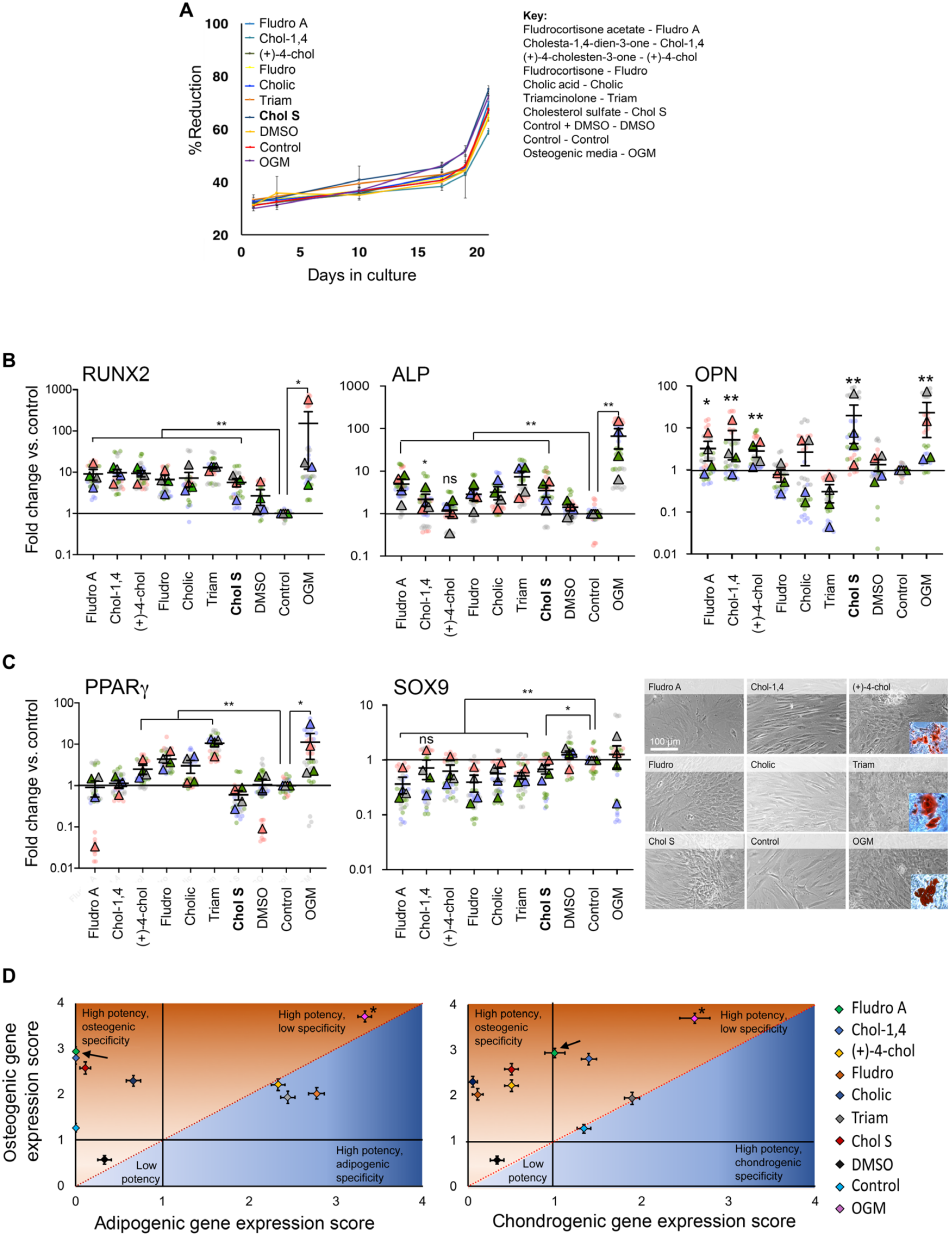
Screening cholesterol sulfate analogs for osteogenic bioactivity in MSCs. (**A**) alamarBlue analysis of MSC metabolic viability with selected steroid supplementation at 1 μM. Results are means ± SEM, *d* = 2, *r* = 2, and *t* = 3, one-way ANOVA with Geisser-Greenhouse correction and Tukey multiple comparison test. (**B**) qRT-PCR comparing osteogenic gene expression in MSCs stimulated with metabolite analogs (1 μM) for 21 days. Fludrocortisone acetate induced osteogenesis at levels similar to OGM. Results are means ± SEM compared to unstimulated day 21 controls, *d* = 4, *r* = 3, *t* = 3; different colors indicate donors, dots indicate individual values, and triangles indicate means; Brown-Forsythe and Welch ANOVA. *P < 0.05 and **P < 0.01. (**C**) Adipogenic (*PPAR*γ) and chondrogenic (*SOX9*) gene expression after 21 days (qRT-PCR) (left). DEX (OGM), (+)-4-cholesten-3-one, and triamcinolone induced adipogenic differentiation. Results are means ± SEM compared to unstimulated day 21 MSCs, *d* = 4, *r* = 3, and *t* = 3, Brown-Forsythe and Welch ANOVA. *P < 0.05 and **P < 0.01. Right, Oil Red O staining of lipid vesicles observed in (+)-4-cholesten-3-one–, triamcinolone-, and OGM-stimulated MSCs. (**D**) Specificity versus potency plot of bioactive metabolites. On the basis of osteogenic, adipogenic, and chondrogenic gene expression, differentiation potency was scored out of 5. Fludrocortisone acetate scored highly for potency and specificity (arrows denote fludrocortisone acetate, and asterisks denote OGM). Control is unstimulated day 21 MSCs. Results are means ± SEM, *d* = 4, *r* = 3, and *t* = 3.

As a first step, we assessed whether the candidate molecules we selected in our screen affected MSC viability. We observed no difference in cell viability between treated cells and unstimulated controls over 2 weeks of culture (Fig. 4A). Next, we performed qRT-PCR to assess the expression of osteogenic markers (*RUNX2*, *ALP*, and *OPN*; Fig. 4B) and of two off-target adipogenic (*PPAR*γ) and chondrogenic (*SOX9*) markers. Several small molecules, most notably fludrocortisone acetate, robustly induced osteogenesis without inducing adipogenesis or chondrogenesis. By contrast, dexamethasone-containing OGM induced both osteogenesis and adipogenesis, as did (+)-4-cholesten-3-one and triamcinolone, indicating their lack of specificity. This off-target induction of adipogenesis was visible in treated cells in the form of accumulating lipid droplets, which were stained by Oil Red O, identifying them as mature adipocytes (Fig. 4C). It is notable that while no candidate molecule induced chondrogenesis, a number, including fludrocortisone acetate, reduced SOX9 expression (Fig. 4C).

To ensure specificity, we checked the candidate molecules with a nonosteogenic, fibroblast cell line. Looking at transcripts of *RUNX2*, *OPN*, *OCN*, *ALP*, *SOX9*, and *PPAR*γ as a panel of osteogenic and other mesenchymal markers, it was seen that all candidate molecules gave no response in the fibroblasts except for fludrocortisone (noting that fludrocortisone acetate gave no change) and dexamethasone-containing OGM that both up-regulated *ALP* (fig. S9). Dexamethasone has been implicated in increasing *ALP* expression in fibroblasts (*38*), again demonstrating the general rather than specific nature of this potent glucocorticoid.

Potency/specificity plots for osteogenesis versus adipogenesis (Fig. 4D) and for osteogenesis versus chondrogenesis (Fig. 4E) show that OGM has high potency but low specificity, while cholesterol sulfate has good potency and high specificity. The synthetic mineralocorticoid, fludrocortisone acetate, however, demonstrated both high potency and high specificity. Protein-level quantitative and qualitative data on the osteogenic potency of the candidate small molecules are provided in fig. S10 and show that fludrocortisone acetate produced high levels of osteogenic marker expression and matrix mineralization.

Although fludrocortisone acetate is known primarily as a mineralocorticoid, it also has a pronounced glucocorticoid effect, approximately one-third that of the widely used glucocorticoid, dexamethasone (*39*, *40*). However, little information is available on its osteogenic potential, although a previous small-molecule screen did identify fludrocortisone acetate as a hit for osteoinduction but provided no insight on its specificity or mechanism (*41*).

### Fludrocortisone acetate and dexamethasone act differently

**Fig. 5:**
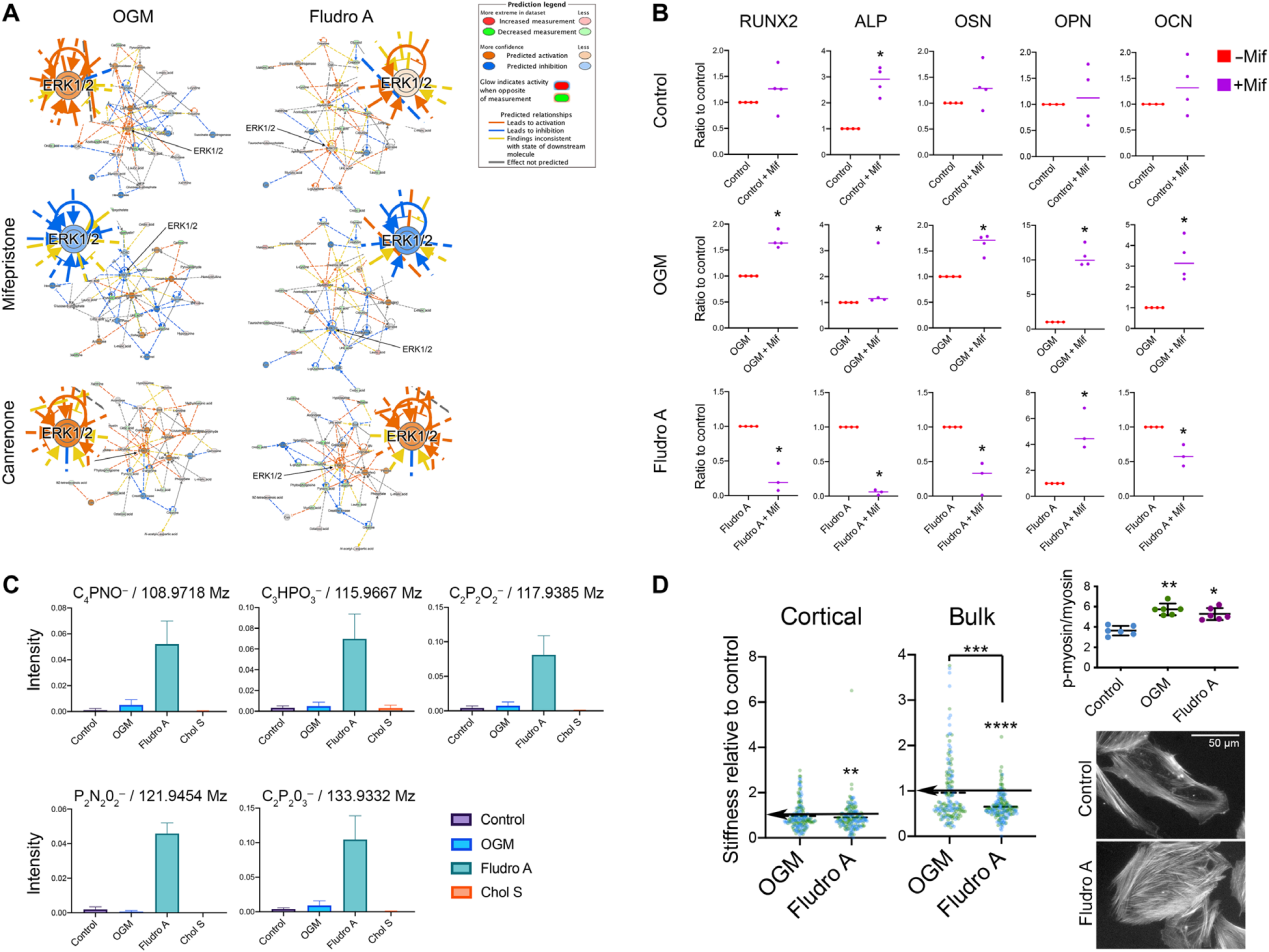
Dexamethasone and fludrocortisone acetate exhibit different osteogenic activities. (**A**) Effects of glucocorticoid (mifepristone) and mineralocorticoid inhibition (canrenone) on top-ranked metabolite-driven biochemical networks. IPA predicts ERK1/2 activation with OGM and fludrocortisone acetate and ERK1/2 hub down-regulation with glucocorticoid inhibition. Mineralocorticoid inhibition caused no change in predicted ERK1/2 activity. Results are means, *d* = 1 and *r* = 4. (**B**) Glucocorticoid inhibition resulted in few osteogenic marker changes in unstimulated day 21 controls. OGM up-regulated all tested markers with mifepristone. Fludrocortisone acetate down-regulated most osteogenic markers with mifepristone. Results are means, *d* = 1, *r* = 4, and *t* = 2, Mann-Whitney *t* test. (**C**) 3D OrbiSIMS of putative breakdown products, showing several unique to fludrocortisone acetate (*d* = 1 and *r* = 3). (**D**) MSC stiffness (Young’s modulus, nanoindentation) in control, OGM, and fludrocortisone acetate groups. Fludrocortisone acetate reduces cell stiffness, *d* = 2, *r* = 2, and *t* ≥100; colors indicate donors, dashed lines indicate means; Kruskal-Wallis with Dunn’s multiple comparison test, significance versus control unless denoted (arrows denote control mean). Analysis of p-myosin/total myosin shows increased intracellular tension with OGM and fludrocortisone acetate treatment compared to unstimulated control. Results are means ± SD, *d* = 1 and *r* = 6; Kruskal-Wallis with Dunn’s multiple comparison test. Fluorescent actin images of control and fludrocortisone acetate MSCs. More organized stress fibers were observed with fludrocortisone acetate versus control. *P < 0.05, **P < 0.01, ***P < 0.001, and ****P < 0.0001.

Next, we treated MSCs with inhibitors of glucocorticoids (mifepristone, also known as RU-486) and mineralocorticoids (canrenone) and performed untargeted metabolomic screens after 7 days of culture with either OGM or fludrocortisone acetate. The resulting data were analyzed by IPA and were compared to control data, with the activity predictor tool enabled to providing biochemical hub information. In the top-ranked network results, around ERK1/2 [which is known to be critical for initiating osteogenesis (*25*–*28*)], little difference was seen between cells cultured in dexamethasone-containing OGM and fludrocortisone acetate (Fig. 5A). Under both OGM and fludrocortisone acetate conditions, MSCs were predicted to up-regulate the ERK1/2 pathway, as expected, in agreement with the OGM and nanovibrational data reported in Fig. 2C. Glucocorticoid signaling inhibition was predicted to lead to the down-regulation of ERK1/2, indicating that the glucocorticoid activity of dexamethasone and of fludrocortisone acetate is likely contributing to their actions. Mineralocorticoid signaling inhibition produced no change in predicted ERK1/2 activity, compared to standard conditions without the inhibitor. This indicates that mineralocorticoid activity is not a driving factor.

Following these results, we cultured MSCs for 3 weeks with mifepristone and assayed for osteogenic marker expression with qRT-PCR (Fig. 5B). Subtle changes in marker expression were observed in treated control cells, while in OGM- and fludrocortisone acetate–stimulated MSCs, large effects were seen on each tested marker’s expression in the presence of glucocorticoid signaling inhibition. In cells cultured with OGM, glucocorticoid signaling inhibition up-regulated osteogenic marker expression. For cells cultured with fludrocortisone acetate, the opposite was true, and inhibition caused the markers’ down-regulation. We speculate that could be because the very high glucocorticoid activity of dexamethasone inhibits its full potential in terms of osteogenesis.

As before, we used 3D OrbiSIMS to investigate the location of fludrocortisone acetate and dexamethasone in the cells. Unlike cholesterol sulfate, neither were identified as remaining in the cells after 3 days of culture (fig. S11). However, fludrocortisone acetate appears to be metabolized quite differently to dexamethasone, with a range of distinctive chemical signatures being noted following fludrocortisone acetate stimulation (Fig. 5C).

We measured Young’s modulus (*E*) of MSCs under control, OGM-, and fludrocortisone acetate–containing conditions and observed a reduction in cortical and bulk E, but only for fludrocortisone acetate–treated cells (Fig. 5D). The difference, however, for fludrocortisone acetate was not as great as that seen with cholesterol sulfate (Fig. 3C). However, intracellular tension and actin cytoskeletal organization were increased in fludrocortisone acetate– and OGM-treated MSCs compared to control (Fig. 5D and fig. S7). It is interesting that fludrocortisone acetate–treated MSCs are less stiff, similarly to cholesterol sulfate–treated cells, while cytoskeletal tension is increased, more similarly to dexamethasone-treated cells.

## DISCUSSION

Our results show that nanovibrational stimulation can be used to identify bioactive metabolites that induce the highly specific osteogenic differentiation of MSCs. The specificity with which osteogenic differentiation is induced is important for overcoming the artifacts caused by using soluble factors, such as dexamethasone (*16*) and BMP2, which induce the differentiation of other lineages, in addition to osteogenesis (*42*, *43*). Had we used dexamethasone to induce osteogenesis in MSC culture, rather than nanovibration, our data show that we would have likely generated many false-positive hits in the metabolome analysis, since many off-target metabolites were differentially regulated.

We also demonstrate that identified bioactive metabolites can be used to investigate structure-function relationships. Our results show that the small molecule, fludrocortisone acetate, which shares structural similarities to the bioactive metabolite we identify here as having osteogenesis-inducing properties, cholesterol sulfate, induces highly specific osteogenic differentiation but with greater potency relative to the activities of cholesterol sulfate and dexamethasone. Fludrocortisone acetate–treated MSCs responded similarly to dexamethasone-treated MSCs, in that both compounds were metabolized rather than retained. However, fludrocortisone acetate– and dexamethasone-treated MSCs showed different glucocorticoid activities and metabolized each compound differently, as monitored by 3D OrbiSIMS. Fludrocortisone acetate also produced similar effects as cholesterol sulfate in terms of the mechanical properties of the cells.

The cytoskeleton/cell stiffness data are interesting as it gives similarities between dexamethasone and fludrocortisone acetate in terms of increased cytoskeletal tension and then it gives similarities between cholesterol sulfate and fludrocortisone acetate in terms of decreased cell stiffness. Within the literature are publications that show decreasing cell stiffness with osteocommitment (*44*, *45*). In these reports, osteogenesis is initiated using dexamethasone (*44*, *45*), which, while always showing a trend of decreasing stiffness, did not greatly lower osteoblast cortical or bulk stiffness in our study. A hypothesis we can allude to from the literature is that typically MSCs have more, but smaller, adhesions than osteoblasts (*46*) and, in MSCs, the cell membrane is more tethered to adhesions reflecting that MSCs need to respond to environmental cues (*45*). Osteoblasts, on the other hand, in common with other connective tissue cells, are required to withstand the loads mechanical strain places on tissues and so tether the membrane to linker complexes such as the ERM proteins (ezrin, radixin, and moesin) (*45*). We have previously reported increased expression of ezrin with onset of MSC osteogenesis (*47*), and other studies have shown that ERM depletion leads to inability to chemically induce osteogenesis (*48*). Thus, while it may seem counterintuitive that MSCs soften as they increase intracellular tension to commit to osteogenesis (as we see with p-myosin), how adhesions and the cytoskeleton interact with the membrane may change to give physiological advantage, and significant softening of the cells could be a useful indicator of specific osteoinducers. In parallel, observation of increased cytoskeletal tension is required to identify potent osteoinducers. This second measure is important as it appears that cholesterol sulfate was a less potent osteoinducer than fludrocortisone acetate as, while it affected membrane stiffness, it did not increase cytoskeletal tension.

Note that while cortical stiffness may decrease with osteocommitment, membrane attachment strength increases. A report that studied membrane properties using micropipette aspiration to measure the critical pressure required for membrane detachment and blebbing during chondrogenic differentiation of MSCs noted increased ERM expression was directly proportional to increased critical pressure (*49*).

That there are differences in metabolism of cholesterol sulfate, dexamethasone, and fludrocortisone acetate is clear. However, understanding target and mechanism will be more challenging. Looking at flux using heavy-labeled isotopes (*50*) and using, for example, chemical proteomics (*51*) where the metabolite is immobilized to a bead and then exposed to cell lysate before mass spectrometry could be sensible next steps.

Note that our activity metabolite testing data have been performed on cells in 2D, and there will be differences when scaled to 3D. For nanovibrational MSC stimulation, there are similarities in known mechanism in 2D and 3D in that intracellular tension from adhesion and mechanoresponsive channels (e.g., transient receptor potential cation and piezo channels) have been identified under both conditions (*7*, *15*, *52*). However, in 2D, the bias appears to be toward adhesion and tension regulation of MSC osteogenesis, and in 3D, the bias is toward mechanoresponsive channel regulation (*7*, *15*, *52*). In the future, it will be interesting to see how these bioactive metabolites influence 3D and 2D cell microenvironments.

By further researching mechanism, the potential to develop new drugs from bioactive metabolites will increase through development of simple in vitro assays. Here, we show ability to select highly specific candidates and then tune potency compared to use of standard protocols. Further, we demonstrate that stiffness and cell tension give good indication of specificity and potency.

Reducing the rate of false-positive results is of critical importance for drug discovery pipelines. Although genomic/high-throughput technology has led to a volume-based research approach, productivity has remained static (*53*). For example, from 2011 to 2016, only 66% of small-molecule projects failed, and critically, only 23% failed before clinical trial (i.e., were “cheap fails”) (*53*). Thus, too many leads make it to trial, and this is largely linked to the use of cell culture models that are not predictive ahead of reliance on nonhuman in vivo models (*54*). Providing more stringent selectivity for the leads entering the pipeline would thus represent a large advantage. We note that we provide proof of concept in MSCs, but this could just as well apply to other stem cell or fastidious cell types.

The candidate small molecule, fludrocortisone acetate, that we identify here is interesting as it is already used as a drug to treat adrenogenital syndrome, postural hypotension, and adrenal insufficiency (*55*). This means that the use for control of stem cells in, for example, osteoporosis could represent a repurposing and thus a simpler regulatory route.

Our work strongly advocates for the use of physical principles to control biology in discovery pipelines. As demonstrated here, approaches such as the use of nanovibrational stimulation can be more specific and more potent than traditional approaches and thus enable identification of highly bioactive metabolites. With a growing physical science toolbox (bioreactors, materials, nanoparticles, etc.) available to researchers, the potential for novel methods for enabling metabolite-based drug discovery is huge.

## MATERIALS AND METHODS

### Nanovibrational apparatus

The design of the nanovibrational bioreactor has been previously described (*7*). Briefly, standard cell culture plates (Corning, NY) were magnetically attached (NeoFlex Flexible Neodymium Magnetic Sheet, 3M, MN, USA) to the vibration plate (dimensions, 128 mm by 176 mm). The vibration plate was secured on its underside to an array of low-profile, multilayer piezo actuators (NAC2022, Noliac A/S CTS, Denmark). To power the piezo array, a custom power supply unit was used, as detailed in a previous publication (*56*), consisting of a signal generator integrated circuit (AD9833, Analog Devices, MA, USA) to provide a 1000-Hz sine wave modulation. A parallel configuration of class AB audio amplifiers (TDA7293, STMicroelectronics, Geneva, Switzerland) was used to amplify the sine wave signal. This results in the vibration plate oscillating at an amplitude of 30 nm and 1000-Hz frequency.

### Interferometric measurement

Vibrational amplitude was measured using a laser interferometry system previously used to accurately measure nanoscale displacements generated by the bioreactor platform used here (*7*, *56*). A USB interferometer (Model SP-S, SIOS Messtechnik GmbH, Ilmenau, Germany) was mounted on a frame, with the laser aimed downward at the measurement site. To measure displacement in 2D cultures, self-adhesive reflective tape was stuck to measurement sites on plastic culture plates to reflect the laser for accuracy. Similarly, to measure displacement in 3D cultures, prismatic tape was adhered to the surface of type I collagen gels. The analysis of the interference pattern between the reflected laser light and the reference signal in the interferometer’s INFAS software (where the time series interference signal is converted to frequency space by fast Fourier transform) allowed the displacement of the target surface to be determined from the produced frequency spectrum. This model of interferometer is sensitive to displacements of 0.1 nm. However, seismic noise (produced by people walking and moving around near the apparatus) can reduce this sensitivity. To prevent this noise from affecting the measurements, the interferometric apparatus was mounted on an optical bench supported by polystyrene blocks to provide noise dampening. For 2D and 3D comparisons of nanovibrational amplitude, 65 measurements were taken at multiple locations on the plates.

### Rheology measurements

Rheological measurements were carried out using an Anton Paar 301 rheometer. Strain sweeps were carried out using a parallel plate system with a 25-mm sand-blasted plate and a gap of 2.8 mm. Two-milliliter collagen gels were prepared beforehand in a 12-well plate and then transferred to the rheometer plate for measuring. Strain sweep tests were performed at an angular frequency of 10 rad s^−1^ and a strain of 0.1 to 5000%. All experiments were performed at 25°C.

#### Calculation of Young’s modulus

An average of the storage shear modulus was taken within the viscoelastic region (0.1 to 10% strain) giving 13.7 Pa. Collagen gels are homogeneous and isotropic from the mechanical point of view, and a reference estimate for the Young’s modulus in the linear region can be obtained from the shear measurements using the following equation

$$G\prime =\frac{E\prime }{2(1+v)}$$

where *G′* is the storage shear modulus, *v* is the Poisson’s ratio, and *E*′ is the Young’s modulus. Assuming full incompressibility for the material (Poisson’s ratio, 0.5), this gives a value of 41.7 Pa for the Young’s modulus

$$G*=\frac{E}{2(1+v)}$$

At ~200% strain, some slipping was observed, and this explains the increased size of the error bars in the region beyond this point.

### Application of nanovibration in 2D and 3D culture

Stro1^+^ MSCs were isolated from the human bone marrow (*10*). MSCs were cultured in expansion media {Dulbecco’s modified Eagle’s medium (DMEM; Sigma-Aldrich), 10% fetal bovine serum (FBS; Sigma-Aldrich), 1% sodium pyruvate (11 mg ml^−1^; Sigma-Aldrich), 1% Gibco MEM nonessential amino acids (Thermo Fisher Scientific), 2% antibiotics [penicillin-streptomycin (6.74 U ml^−1^; Sigma-Aldrich) and fungizone (0.2 μg ml^−1^; Sigma-Aldrich)]} or in osteogenic differentiation media [OGM; DMEM expansion media, supplemented with 100-μmol ascorbic acid (Sigma-Aldrich), 100-nmol dexamethasone (Sigma-Aldrich), and 10-mmol glycerol phosphate (Sigma-Aldrich)]. Nanovibration (30-nm displacement; 1000 Hz) was applied to MSCs in 2D and 3D culture. Nanovibrated cell responses were compared to those of unstimulated control MSCs cultured in expansion media and to those of MSCs cultured in osteogenic differentiation media without nanovibration.

In 2D culture, cells were seeded at 4 × 10^3^ cells cm^−2^ in standard cell culture plates in either expansion or OGM. For 3D culture, type I collagen gel was prepared by the addition of 10× DMEM (Sigma-Aldrich), FBS, expansion media, and rat-tail type I collagen (2.05 mg ml^−1^; First Link) in 0.16% acetic acid. The pH of the collagen solution was neutralized through the addition of 0.1 M NaOH on ice until a constant pH 7 was reached. The appropriate number of MSCs was then added to give a cell density of 4 × 10^4^ cells ml^−1^, and the solution was mixed by pipette to provide a homogenous cell suspension. Solutions were pipetted into culture 24-well plates (1 ml of gel/cell solution giving a depth of 5.2 mm) and allowed to gel in humidified incubators [37°C and 5% (v/v) CO_2_] for 2 hours. Subsequently, wells were flooded with the relevant media. Plates containing cells for nanovibration were then magnetically attached to the nanovibration bioreactor in cell culture incubators.

### Effect of nanovibration or OGM on MSC differentiation

To compare the effects of nanovibration or OGM on MSC osteogenic differentiation, cells were seeded as above under 2D and 3D culture conditions. Over a 28-day time course, cells were stimulated continuously either with nanovibration (30 nm/1000 Hz) or with OGM or were left unstimulated, as controls. Samples were taken for qRT-PCR at days 0, 7, 10, 14, 21, and 28 to determine changes in the expression of early (*RUNX2*; *OSX*) and late (*OPN*; *OCN*) osteogenic marker genes. To analyze off-target gene expression, the expression of adipogenesis (*PPAR*γ) and chondrogenesis (*SOX9*) markers was also analyzed by qRT-PCR.

### Quantitative reverse transcription polymerase chain reaction

RNA was extracted from 2D and 3D cultures through TRIzol extraction (Life Technologies). Media were removed, and cells were washed in sterile phosphate-buffered saline (PBS) on ice. Equal volumes of TRIzol reagent were added to cells, and cells were incubated for 10 min at room temperature. TRIzol was transferred to 1.5-ml tubes, and 0.2 ml of chloroform was added to each tube per 1 ml of TRIzol. TRIzol/chloroform solutions were vortexed and centrifuged (13,000*g*/4°C). Following centrifugation, the upper aqueous layer was transferred to a new 1.5-ml tube and an equal volume of 70% (v/v) ethanol added. This solution was mixed by repeated inversion of the tubes. RNA was then extracted from this solution using the QIAGEN RNeasy Extraction Kit (including deoxyribonuclease step), according to the manufacturer’s instructions. RNA was eluted in nuclease-free water and quantified using the NanoDrop and normalized across all samples. Complementary DNA (cDNA) (1000 ng per sample) was prepared by reverse transcription using the QIAGEN QuantiTect Kit, according to the manufacturer’s instructions. cDNA concentration was normalized to 5 ng μl^−1^ by dilution in nuclease-free water. Using the 7500 real-time PCR system from Applied Biosystems, qRT-PCR was performed using the QuantiFast SYBR Green qRT-PCR Kit (QIAGEN) and specific human gene target primers (Eurofins Genomics) (Table 1 and further information in table S1), validated by dissociation/melt curve analysis. Ten nanograms of cDNA was loaded into each qRT-PCR reaction. qRT-PCR products were quantified using the 2^−ΔΔ*C*t^ method (*57*) and normalized to the housekeeping gene glyceraldehyde-3-phosphate dehydrogenase (*GAPDH*), which was confirmed to remain stable under all culture conditions.

**Table 1:** List of primers.

**Gene**	**Forward primer**	**Reverse primer**
*RUNX2*	GGTCAGATGCAGGCGGCCC	TACGTGTGGTAGCGCGTGGC
*OSX*	GGCAAAGCAGGCACAAAGAAAG	AATGAGTGGGAAAAGGGAGGG
*OCN*	CAGCGAGGTAGTGAAGAGACC	TCTGGAGTTTATTTGGGAGCAG
*OPN*	AGCTGGATGACCAGAGTGCT	TGAAATTCATGGCTGTGGAA
*OSN*	AGAATGAGAAGCGCCTGGAG	CTGCCAGTGTACAGGGAAGA
*ALP*	ATGAAGGAAAAGCCAAGCAG	CCACCAAATGTGAAGACGTG
*PPARG*	GACAGGAAAGACAACAGACAAATC	GGGGTGATGTGTTTGAACTTG
*FABP4*	CCTTTAAAAATACTGAGATTTCCTTCA	GGACACCCCCATCTAAGGTT
*GLUT4*	CTGTCCACCAAGCCCTCTC	CATCCCCAGTCTCCACTGTT
*SOX9*	GCTCTGGAGACTTCTGAA	GGTACTTGTAATCCGGGTG
*COL2A1*	GGCTTCCATTTCAGCTATG	CAGTGGTAGGTGATGTTC
*ACAN*	GGCTTCCACCAGTGTGAC	GTGTCTCGGATGCCATACG
*GAPDH*	TCAAGGCTGAGAACGGGAA	TGGGTGGCAGTGATGGCA

### ALP activity assay

To assess ALP activity in cultured cells, a colorimetric assay was used (ab8369, Abcam). This kit uses *p*-nitrophyenyl phosphate (pNPP) as a phosphatase substrate that turns yellow [maximum optical density (OD_max_), 405 nm] when dephosphorylated by ALP. Increased ALP activity in cultured MSCs was indicative of the formation of osteogenic cell phenotypes. The assay was performed according to the manufacturer’s instructions. Briefly, cells were trypsinized, counted, pelleted, and washed in ice-cold PBS. Cells were then resuspended in 50-μl assay buffer per 1 × 10^5^ cells, then homogenized on ice, and centrifuged at 13,000*g* for 15 min at 4°C. The supernatant was transferred to a new tube. Supernatant volume to be added was optimized on the basis of standard curve concentrations, and the reaction volume was adjusted to 80 μl per well. Fifty microliters of 5 nM pNPP solution was added to each well and incubated at 25°C for 60 min protected from light. Stop solution was then added to each well, and an OD of 405 nm was measured on a microplate reader. Corrected mean absorbance values were calculated by subtracting blank readings, and ALP activity was determined by applying the generated standard curve and using the following equation

ALP activity (μmol/min/ml or U/ml)=(B/ΔT*V)*D

where *B* is the amount of pNPP in sample well calculated from standard curve (in micromoles), Δ*T* is the reaction time (in minutes), *V* is the original reaction sample volume (in milliliters), and *D* is the sample dilution factor.

### Metabolomics

MSCs were stimulated with nanovibration for 7 and 14 days in 2D and 3D (collagen gels; 2 mg ml^−1^) culture. Nonstimulated samples cultured in expansion media and OGM were used as controls. Metabolites were extracted using a 1:3:1 chloroform/methanol/water extraction buffer and vigorously shaken at 4°C for 1 hour. Following this, metabolite extraction solution was collected, transferred to 1.5-ml tubes, and centrifuged for 3 min at 13,000*g* at 4°C. Metabolomics was performed through hydrophilic interaction LC-MS analysis (UltiMate 3000 RSLC, Thermo Fisher Scientific) with a 150 mm by 4.6 mm ZIC-pHILIC column running at 300 μl min^−1^and Orbitrap Exactive (Thermo Fisher Scientific). A standard pipeline, consisting of XCMS (*58*) (peak picking), MzMatch (*59*) (filtering and grouping), and IDEOM (*60*) (further filtering, postprocessing, and identification), was used to process the raw mass spectrometry data. Identified core metabolites were validated against a panel of unambiguous standards by mass and retention time. Further putative identifications were allotted mass and predicted retention time (*22*). Means and SEs of the mean were generated for every group of picked peaks, and the resulting metabolomics data were uploaded to IPA software for pathway analysis.

### Chemistry

Dexamethasone, cholesterol sulfate, fludrocortisone, fludrocortisone acetate, triamcinolone, cholic acid, and (+)-4-cholesten-3-one were obtained from commercial suppliers and used as received. Cholesta-1,4-dien-3-one that was prepared according to a literature procedure on related steroids (*61*) to a solution of (+)-4-cholesten-3-one (100 mg, 0.26 mmol) in dioxane (1.6 ml) was added *tert*-butyldimethylsilyl chloride (2.0 mg, 0.013 mmol), and then the mixture was cooled to 0°C. To the solidified solution, DDQ was added (66 mg, 0.29 mmol). The mixture was allowed to warm to room temperature and then stirred for 3 days. The solvent was removed in vacuo and the residue dissolved in CH_2_Cl_2_ (40 ml) and then washed with saturated aqueous Na_2_S_2_O_3_ (40 ml), NaHCO_3_ (40 ml), and brine (40 ml). The organic phase was dried over Na_2_SO_4_, filtered, and concentrated in vacuo to give a yellow oil. Purification by flash chromatography (petroleum ether/ethyl acetate, 9:1) afforded the title compound as a white solid (33 mg, 33%). Analytical data (fig. S12) were in accordance with literature values (*62*). ^1^H nuclear magnetic resonance (400 MHz, CDCl_3_) δ: 7.05 parts per million (ppm) (1H, d, *J* = 10.1 Hz), 6.22 ppm (1H, dd, *J* = 10.1, 1.9 Hz), 6.06 ppm (1H, s), 2.50 to 2.42 ppm (1H, m), 2.37 to 2.32 ppm (1H, m), 2.06 to 2.01 ppm (1H, m), 1.96 to 1.79 ppm (2H, m), 1.69 to 1.46 ppm (6H, m), 1.36 to 1.26 ppm (4H, m), 1.23 ppm (3H, s), 1.20 to 0.97 ppm (9H, m), 0.90 ppm (3H, d, *J* = 6.5 Hz), 0.86 ppm (6H, dd, *J* = 6.6, 1.8 Hz), and 0.74 ppm (3H, s).

### Culture with cholesterol sulfate and selected compounds

To assess the bioactivity of the steroid library, MSCs were seeded at 4 × 10^3^ cells cm^−2^ in standard cell culture plates and allowed to attach overnight [37°C and 5% (v/v) CO_2_]. Media were then exchanged for media supplemented with the relevant small molecule at 0.1, 1, or 10 μM. Concentration were selected for bioactivity screening by qRT-PCR after 21 days of culture by assessing *RUNX2* expression. Through this, 1 μM concentrations were selected for further experimental comparisons in culture time courses of up to 21 days. The relevant compound was supplemented to control expansion media before media changes, which were performed every other day.

### alamarBlue assay

At determined intervals during culture, cell culture media were removed, and cells washed with prewarmed, sterile PBS. alamarBlue resazurin (10%, v/v; Bio-Rad) was diluted in phenol-red free media (D5030, Sigma-Aldrich) and added to each hydrogel. Cells were incubated in alamarBlue working solution for 4 hours [at 37°C and 5% (v/v) CO_2_]. After incubation, supernatant was transferred to 96-well plates and absorbances read at 570 and 600 nm to determine the metabolism of alamarBlue. The percentage of alamarBlue reduction was calculated as follows

%reduction of Alamar Blue=((O2×A1)–(O1×A2)/(R1×N2)−(R2×N1))×100

where O1 and O2 are the molar extinction coefficients of oxidized alamarBlue at wavelengths of 570 and 600 nm, respectively. R1 and R2 are the molar extinction coefficients of reduced alamarBlue at wavelengths of 570 and 600 nm, respectively. A1 and A2 are the observed absorbance readings for test wells at wavelengths of 570 and 600 nm, respectively. N1 and N2 are the observed absorbance readings for the negative control wells at wavelengths of 570 and 600 nm, respectively.

### Bioactive compound specificity

To determine the osteogenic specificity of bioactive compounds, a ranking system was developed and used. Osteogenic (*RUNX2*, *OSX*, *ALP*, and *OPN*), adipogenic [*PPAR*γ, fatty acid–binding protein 4 (*FABP4*), and glucose transporter type 4 (*GLUT4*)], and chondrogenic [*SOX9*, *ACAN* (aggrecan), and *COL2A1* (type II collagen, alpha 1)] gene expression was used to determine cell differentiation along each lineage. Fold change gene expression after stimulation with each compound for 21 days was determined at 1 μM concentration, and fold changes were grouped and scored as follows: fold changes 1 to 2, 1 point; 2 to 5, 2 points; 5 to 10, 3 points; 10 to 20, 4 points; more than 20, 5 points. Scores for each gene were recorded, and mean values for each category and each metabolite were calculated out of 5. These scores were then plotted against each other in pairs to determine a relative osteogenic versus chondrogenic and osteogenic versus adipogenic gene expression induction, providing information about the potency and specificity of small-molecule action.

### Pathway inhibition

To assess the target specificity of the glucocorticoid and mineralocorticoid stimulation, we used the inhibitors mifepristone (M8046, Sigma-Aldrich) and canrenone (SML 1497, Sigma-Aldrich), respectively. Cells were seeded at 2 × 10^3^ cells cm^−2^ for the specified duration of experiments (7 days for the metabolomics experiments and 3 weeks to assess the long-term effect of mifepristone-induced glucocorticoid inhibition on osteogenic marker expression). Mifepristone was used at 10 μM, and canrenone was used at 100 μM, and they were supplemented in the medium with every feed (twice per week) for the duration of each experiment.

### 3D OrbiSIMS

3D chemical image analysis of the sample series was performed using dual-beam [mode 9 (*35*)] TOF spectrometry, using a 30-keV Bi_3_^+^ primary ion source (0.3-pA target current) and a 10-keV Ar_1450_^+^ sputter ion source (3-nA target current). A sputter crater of 400 μm by 400 μm was etched with the central 200 μm by 200 μm area analyzed at a resolution of 256 pixels by 256 pixels. In each case, >3 cells were analyzed per sample area. Cells were also depth profiled using single beam Orbitrap analysis [mode 4 (*35*)] to acquire relatively high-resolution mass spectrometry data (>240,000) for the sample series. In this case, a 20-keV Ar_3000_^+^ primary ion source (240-pA target current) was used with a sputter crater of 284 μm by 284 μm with the central 200 μm by 200 μm area analyzed. Three analytical repeat areas were analyzed for each sample. A random raster function was applied throughout, as well as charge compensation with the application of a low-energy electron floodgun.

### Single-cell force spectroscopy

Single-cell mechanics were evaluated using a nanoindentation device (Chiaro, Optics11, Amsterdam, NL) mounted on top of an inverted phase contrast microscope (Evos XL Core, Thermo Fisher Scientific, Paisley, UK) following a previously described approach (*63*). Human MSCs were left to incubate for 72 hours at 37°C and 5% CO_2_ with the corresponding media (basal media, OGM, and basal media with metabolites). They were then washed once with basal media only before the measurement began. All measurements were acquired at room temperature, keeping the measuring time under 90 min, to avoid changes to the cells’ mechanical properties that are associated with cell degeneration. A total of 35 cells from two biological replicates were measured for each condition. The selected cantilever had a stiffness of 0.032 N m^−1^ and held a spherical tip of 3.25-μm radius (serial number P190610). A tight three-by-three map with 500-nm spacing was acquired (total of nine indentations), aiming at the cellular soma (above the nucleus). Single indentations were acquired at the same speed of 2 μm s^−1^, exploiting the whole range of the vertical actuator, 10 μm. After every experiment, the probe was washed in 70% ethanol for 10 min.

The collected curves were bulk analyzed using a custom software programmed with Python 3 (Python Software Foundation, www.python.org) and the Numpy/Scipy Scientific Computing Stack (*64*). Curves were first aligned using a baseline detection method based on the histogram of the force signal (*65*), and the corresponding indentation was calculated for each curve. To quantify the mechanical properties, data were fitted with the Hertz model (*66*). While the hypothesis behind the theoretical derivation of the Hertz formula (isotropy, homogeneity, and pure elasticity of the sample) are fairly satisfied by a cellular system, it has been shown that the corresponding Young’s modulus can provide a robust indicator of the elasticity if the experimental procedure is carefully designed (*67*). To ensure consistency of the results, all the experimental parameters were kept constant during an experimental session for all different conditions (in particular, the same probe and calibration were used), and the results were reported, indicating changes of elasticity relative to the control. The average absolute value for the control was also reported, but relative changes were typically more reliable and meaningful (*67*).

The calculation of the Young’s modulus of single cells based on nanoindentation experiments strongly depends on the indentation depth of the corresponding measurement. This effect is partially due to artifacts such as the finite thickness of the sample (*68*) and the parabolic approximation for the calculation of the Hertz formula (*69*). Keeping the maximum indentation used in the calculation under ~10 to 15% of the thickness, and 20 to 25% of the radius of the indenter, is a rule of thumb typically used in literature (in our case, 600 to 700 nm would match these requirements). Nevertheless, for indentations lower than this threshold, a trend in the measured Young’s modulus as a function of the indentation depth appears, which is associated with the inhomogeneity of the cell (*70*). Here, we exploited this approach, trying to isolate the elasticity of the cortical region from the bulk of the cell. The actomyosin cortex is a very thin network of cytoskeletal elements that lies directly beneath the plasma membrane and is present in all mammalian cells (*71*). The thickness of this rigid and compact structure challenges current microscopy approaches, and a precise measurement is often complex, but existing estimates typically range between 200 and 300 nm (*72*). In this work, we selected an indentation depth of 270 nm to identify the cortical region. We called “cortical elasticity” the value of the Young’s modulus that was obtained by evaluating all the indentation curves up to this threshold. Similarly, we called “bulk elasticity” the value of the Young’s modulus that was calculated up to an indentation of 640 nm (10% of the tip diameter, the maximum to remain in the Hertzian regime).

### In-cell western assays

MSCs in 24-well plates were fixed using 10% (v/v) formaldehyde for 20 min at room temperature. Cells were then permeabilized with 0.1% (v/v) Triton X-100 in PBS for 10 min at room temperature and blocked using 1% milk protein in PBS with 0.1% (v/v) Tween 20 (PBST). Primary antibodies to target proteins diluted in blocking buffer (1:200) were incubated with cells overnight at 4°C with gentle agitation (Table 2). After incubation, cells were washed five times with PBST. As normalization controls, CellTag 700 stain (LI-COR) was diluted in blocking buffer (1:1000). To this solution, the relevant secondary antibodies were added (1:2000; LI-COR). Cells were incubated with this solution for 1.5 hours at room temperature with gentle agitation, followed by five washes with PBST. Quantitative spectroscopic scanning and analysis were carried out using the LI-COR Odyssey Sa. All dyes and secondary antibodies were purchased from LI-COR. For analysis, internally normalized fluorescent intensities were normalized against unstimulated controls to generate fold change fluorescent intensities.

**Table 2:** List of primary antibodies.

**Target**	**Company**	**Catalog number**
RUNX2	Santa Cruz Biotechnology	Sc-390351
Osteonectin	Millipore	AB1858
OPN	Santa Cruz Biotechnology	Sc-21742
OSX	Santa Cruz Biotechnology	Sc-393325
ALP	Abcam	Ab354
OCN	Santa Cruz Biotechnology	Sc-365797
Total myosin	Cell Signaling Technology	3672s
P (S19) myosin light chain	Cell Signaling Technology	3675s

### Immunocytochemistry

Cells were fixed in 10% (v/v) formaldehyde/PBS at 4°C for 1 hour. Cells were permeabilized with 0.1% (v/v) Triton X-100 in PBS for 10 min at room temperature and blocked with 1% (w/v) bovine serum albumin in PBS with 0.1% (v/v) Tween 20 (PBST) for 1 hour at room temperature. Following blocking, the relevant primary antibodies (Table 2) were incubated with cells in blocking buffer overnight at 4°C. Cells were washed three times in PBST and incubated with biotinylated secondary antibodies in blocking buffer (1:50; Vector Laboratories) for 1 hour at room temperature. Cells were again washed three times in PBST and incubated with fluorescein isothiocyanate– or Texas Red–conjugated streptavidin in blocking buffer (1:50; Vector Laboratories). Where appropriate, cell F-actin was labeled through 1-hour incubation at room temperature with rhodamine-conjugated phalloidin (1:1000 in blocking buffer). Nuclei were stained using VECTASHIELD mountant with 4′,6-diamidino-2-phenylindole nuclear stain (Vector Laboratories).

### Histological staining

#### Oil Red O

Cells were fixed in 10% (v/v) formaldehyde/PBS at 4°C for 1 hour, then washed with distilled water three times, and rinsed with 60% (v/v) isopropanol. Oil Red O solution was then added to the cells, and cells were incubated at room temperature for 15 min. Dye solution was removed, and cells were washed again with 60% (v/v) isopropanol, washed three times in distilled water, and imaged on an inverted microscope (Olympus, PA, USA) operated through Surveyor software (v.9.0.1.4, Objective Imaging, Cambridge, UK). Images were processed using ImageJ [v.1.50g, National Institutes of Health (NIH), USA].

#### Alizarin Red staining

Cells were fixed in 10% (v/v) formaldehyde/PBS at 4°C for 1 hour. After washing with PBS, fixed cells were stained with 2% (w/v) Alizarin Red solution (pH 4.1 to pH 4.3) for 15 min at room temperature. After staining, cells were washed in deionized water and imaged on an inverted microscope (Olympus, Pennsylvania, USA), operated through Surveyor software (v.9.0.1.4, Objective Imaging, Cambridge, UK). Images were processed using ImageJ (v.1.50g, NIH, USA).

### Statistics

Statistical analysis of the effects of nanovibration and OGM on osteogenic gene expression through qRT-PCR was performed by one-way analysis of variance (ANOVA) with Holm-Sidak’s multiple comparison test. Data are means ± SEM or means ± SD. Statistical analysis of off-target gene expression induction by nanovibration or OGM was conducted through Kruskal-Wallis with Dunn’s multiple comparison test. ALP assay data were statistically analyzed using Kruskal-Wallis with Dunn’s multiple comparison test, as was the bioactive small molecule–mediated induction of osteogenic genes. alamarBlue experiments were statistically compared by one-way ANOVA with Geisser-Greenhouse correction and Tukey multiple comparison test. All statistical analysis was performed using GraphPad Prism software (v8.0.0; GraphPad Software Inc.)

Please note that we denote replicates as follows: number of donors that were used for the particular experiment (i.e., experimental repeats) = *d*, replicates (i.e., number of wells) = *r*, and technical replicates for quantitative PCR etc. to test pipetting error = *t* (if used). Full information is given in Table 3. Please see Supplementary Methods for further details.

**Table 3:** Replicates used for each experiment. Raw data can be found at (*29*).

**Figure**	**Experiment**	**Cells from *X* number of** **donors**	**Well replicates per group**	**Technical replicates per** **group**
Fig. 1D and fig. S2	Nanovibration/OGM osteogenic gene expression analysis—qRT-PCR	1	4	3
Fig. 1E and fig. S3E	ALP activity assay	3	2	3
Fig. 1F and fig. S3F	Off-target gene expression in nanovibration, OGM- stimulated cells- qRT-PCR	3	3 (pooled)	3
Fig. 2	Metabolomics	1	4	1
Fig. 3A	Metabolite compound osteogenic gene expression—qRT-PCR	1	3	3
Fig. 3C	Nanoindentation	2	2	>100
Fig. 4A	alamarBlue	2	2	3
Fig. 4B	Metabolite compound induction of osteogenic gene expression—qRT-PCR	4	3	3
Fig. 4C	Off-target gene expression effects of compounds	4	3	3
Fig. 4 (D and E)	Off-/on-target gene expression effects of compounds	4	3	3
Fig. 5B	Glucocorticoid receptor inhibition—qRT-PCR	1	4	2
Fig. 5C and fig. S7	3D OrbiSIMS	1	3	3
Fig. 5D	Nanoindentation	2	2	>100
Fig. 5D	Myosin	1	6	1
fig. S8	Actin staining	1	3	1
fig. S10A	In-cell western of osteogenic protein expression compound supplementation	1	2	2
fig. S10B	Immunofluorescent staining	1	2	5
fig. S10C	Colorimetric ALP activity assay	2	5	2
fig. S10D	Alizarin Red staining	3	1	5
fig. S6	Effects of compound concentration—qRT-PCR	4	3	3
fig. S9	Effects of compounds on fibroblasts	1	3	3

## SUPPLEMENTARY MATERIALS

Supplementary material for this article is available at http://advances.sciencemag.org/cgi/content/full/7/9/eabb7921/DC1

## Appendix

View/request a protocol for this paper from *Bio-protocol*.
